# Nanoscale imaging of antiferromagnetic domains in epitaxial films of Cr_2_O_3_*via* scanning diamond magnetic probe microscopy

**DOI:** 10.1039/d2ra06440e

**Published:** 2022-12-20

**Authors:** Adam Erickson, Syed Qamar Abbas Shah, Ather Mahmood, Ilja Fescenko, Rupak Timalsina, Christian Binek, Abdelghani Laraoui

**Affiliations:** Department of Mechanical & Materials Engineering, University of Nebraska-Lincoln 900 N 16th St., W342 NH Lincoln Nebraska 68588 USA alaraoui2@unl.edu; Department of Physics and Astronomy and the Nebraska Center for Materials and Nanoscience, University of Nebraska-Lincoln 855 N 16th St Lincoln Nebraska 68588 USA binek@unl.edu; Laser Center, University of Latvia Jelgavas St 3 Riga LV-1004 Latvia

## Abstract

We report direct imaging of boundary magnetization associated with antiferromagnetic domains in magnetoelectric epitaxial Cr_2_O_3_ thin films using diamond nitrogen vacancy microscopy. We found a correlation between magnetic domain size and structural grain size which we associate with the domain formation process. We performed field cooling, *i.e.*, cooling from above to below the Néel temperature in the presence of a magnetic field, which resulted in the selection of one of the two otherwise degenerate 180° domains. Lifting of such a degeneracy is achievable with a magnetic field alone due to the Zeeman energy of a weak parasitic magnetic moment in Cr_2_O_3_ films that originates from defects and the imbalance of the boundary magnetization of opposing interfaces. This boundary magnetization couples to the antiferromagnetic order parameter enabling selection of its orientation. Nanostructuring the Cr_2_O_3_ film with mesa structures revealed reversible edge magnetic states with the direction of magnetic field during field cooling.

## Abstract 2

We report direct imaging of boundary magnetization associated with antiferromagnetic domains in magnetoelectric epitaxial Cr_2_O_3_ thin films using diamond nitrogen vacancy microscopy.

## Graphical abstract

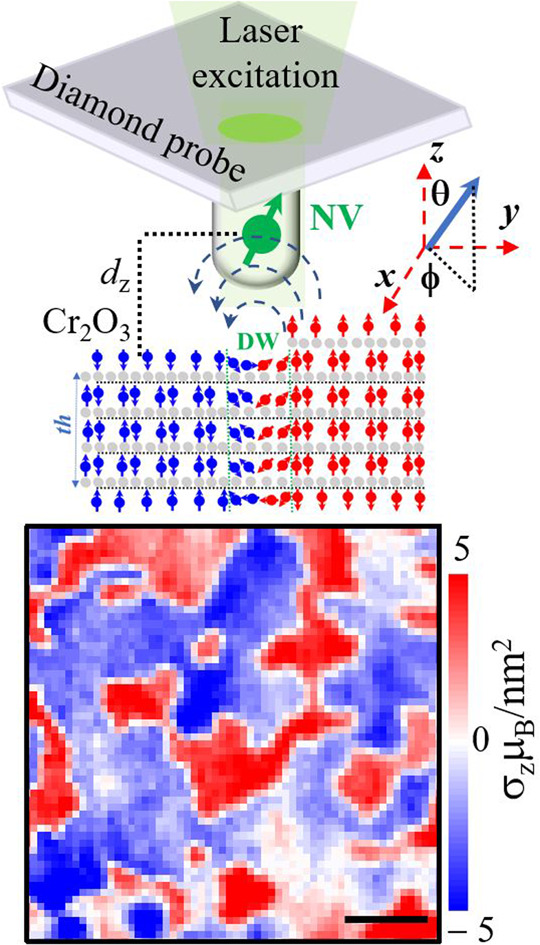

## 1. Introduction

Under zero applied magnetic field and below Néel ordering temperature (*T*_N_) the antiferromagnetic (AFM) interactions in AFM materials lead to a collinear or noncollinear spin orientation in the ground state that can be represented by one single vector of unit length, called the Néel vector.^1^ Because of their zero net magnetization, AFMs were originally considered less useful for spintronic devices. This presumption has been negated by recent discoveries in the electrical control and detection of the orientation of the Néel vector in high *T*_N_ (>300 K) AFM materials such as CuMnAs,^2–4^ Mn_2_Au,^5,6^ and Mn_3_Sn.^7,8^ In addition, these materials are robust against external magnetic fields and display no magnetic stray fields. The potential for ultrafast dynamics in the THz range makes antiferromagnets well suited for miniaturized ultrafast spintronic devices.^9,10^

Magnetoelectric (ME) AFMs have an equilibrium surface or boundary magnetization that couples to the Néel vector and can be controlled by electric field, offering additional means for controlling magnetic order of AFM materials for spintronics devices.^11^ The AFM ME sesquioxide Cr_2_O_3_ (chromia) is an archetypical oxide that permits voltage-control of the Néel vector in the presence of an applied magnetic field.^11,12^ Thin films of Cr_2_O_3_ have been used to realize voltage-control of the peculiar boundary magnetization of single domain ME AFMs, detected through an exchange bias produced by Cr_2_O_3_ on an adjacent ferromagnet CoPd.^12^ A purely antiferromagnetic magnetoelectric random access memory has been realized in Pt(20 nm)/α-Cr_2_O_3_(200 nm)/Pt(2.5 nm) structures with 50-fold reduction of the writing threshold compared with ferromagnet-based materials,^13^ making chromia a potential material to use in AFM spintronics.^14^ Later, magnetic force microscopy (MFM) and photoemission electron microscopy (PEEM) combined with X-ray magnetic circular dichroism (XMCD) were used to spatially map the electrically controlled surface magnetization domain structures of pristine Cr_2_O_3_ films with domain size of few micrometers. However, the MFM and XMCD-PEEM contrast is very small due to the weak stray magnetic field generated from uncompensated spins at the surface of Cr_2_O_3_ film,^15^ and the used techniques tend to suffer from low spatial resolution (*e.g.*, >50 nm in PEEM).^16,17^

Magnetic microscopy based on nitrogen vacancy (NV) centers in diamond has become a versatile tool to study magnetic phenomena at the nanoscale.^18–22^ NV scanning probe magnetometry (NV-SPM) magnetic imaging of 180-degree domains in granular thin films of Cr_2_O_3_ revealed an average magnetic domain size of 230 nm with narrow domain walls^23^ contrary to theoretical expectation based on the weak anisotropy of Cr_2_O_3_.^24^ NV-SPM in combination with second-harmonic-generation microscopy measurements on bulk Cr_2_O_3_ crystals showed that most 180° domain walls are Bloch-like and can coexist with Néel walls in crystals with a high in-plane anisotropy.^25^ The domain wall (DW) width has important implications for antiferromagnetic spintronic device applications. Large DWs limit device scaling and investigations in epitaxial Cr_2_O_3_ films are missing. Here we report NV-SPM imaging of surface AFM domains with DWs and edge domains of nanostructured epitaxial Cr_2_O_3_ (0001) thin films under different experimental (magnetic field, temperature) conditions in thin films grown *via* pulsed laser deposition. We found distinct differences in the magnetic domain formation compared with reports in the literature on samples grown by sputtering methodology.

## 2. Experimental conditions

**Fig. 1:**
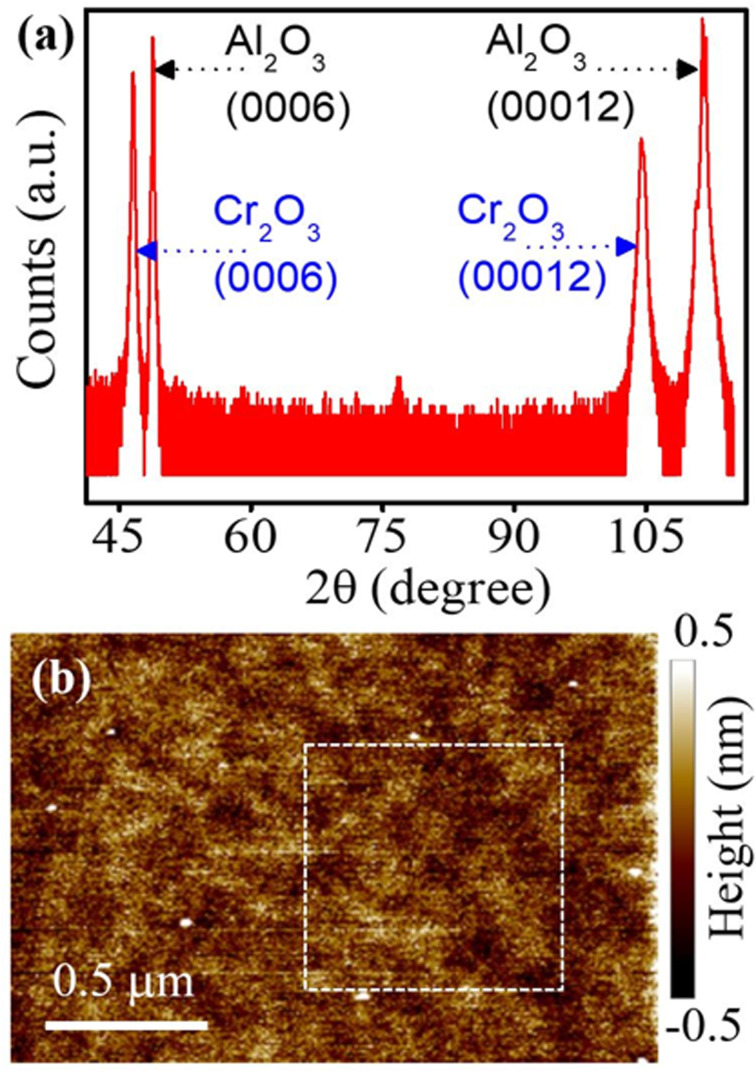
(a) XRD pattern showing the Cr_2_O_3_ (0006) and Al_2_O_3_ (00012) peaks. (b) Atomic force microscopy topography image of the Cr_2_O_3_ (0001) film surface.

The samples used in this study consist of Cr_2_O_3_ films grown on (0001) sapphire substrates. The sapphire substrates were cleaned using a modified Radio Corporation of America protocol.^26^ 200 nm thick Cr_2_O_3_ films were deposited using pulsed laser deposition (PLD).^27^ The substrates were heated to 1073 K during the deposition. A KrF excimer laser with pulse energies of 200 mJ, a spot size of about 6 mm^2^, and a pulse width of 20 ns (at a repetition rate of 10 Hz) was used to ablate the Cr_2_O_3_ target. The target-to-substrate distance was kept at about 9 cm and the substrate rotation rate was at 4 rpm. X-ray diffraction (XRD) measurements (Fig. 1a) revealed the (0001) orientation of Cr_2_O_3_ film. The atomic force microscopy topography map (Fig. 1b) shows the root mean square (RMS) surface roughness value of 0.155 nm (0.13 nm in the dashed square area).

**Fig. 2:**
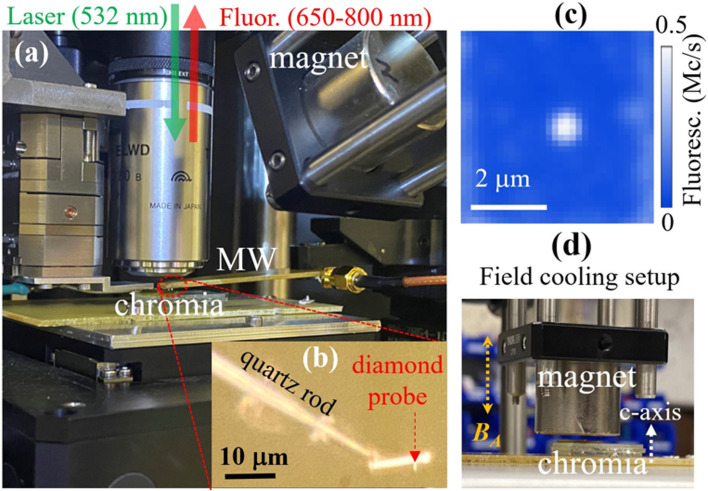
(a) A picture of NV-SPM setup showing 532 nm laser excitation and fluorescence (650–800 nm) detection through a high NA objective. (b) Optical image of the diamond probe attached to a quartz rod. (c) Fluorescence image of NV located ∼12 nm from the diamond probe surface in (b). (d) Field cooling setup with a permanent magnet (up to ± 1 T) mounted on a hot plate (up to 500 K).

The NV-SPM microscope (Fig. 2a) used in this study is a state-of-the-art home built combined platform hosting tuning-fork-based atomic force microscope and optical confocal microscope.^28^ We used a long working distance (4 mm), high NA (=0.8) Nikon objective and a Qnami probe tip. The tuning fork is attached to Attocube *XYZ* step motors with positioning resolution below 1 nm. An additional piezo-based *xyz* scanner, purchased from Npoints, provides a scan range of 100 μm × 100 μm × 20 μm. The special self-sensing and self-actuating SPM sensor is based on a diamond cantilever attached to a quartz rod (Fig. 2d). Fig. 2c shows the fluorescence image of NV center implanted 9 ± 3 nm below the surface of a (100) oriented diamond probe (Fig. 2b). We used SRIM calculations to determine the NV depth for ^15^N implantation of diamond substrate with an energy of 6 keV.^29^ The negatively charged NV center, composed of a substitutional nitrogen near to a vacancy site, is an electronic spin 1 with a spin-triplet (|*m*_s_ = 0>, |*m*_s_ = ±1>) in the ground state. 532 nm laser illumination induces spin-dependent fluorescence (650–800 nm)^18^ allowing optical detected magnetic resonance (ODMR) of its spin state, Fig. 2b. The applied magnetic field, provided by a permanent magnet (up to 30 mT), splits |*m*_s_ = ±1> state *via* Zeeman effect and leads to two (|*m*_s_ = 0> to |*m*_s_ = −1> and |*m*_s_ = 0> to |*m*_s_ = −1>) ODMR peaks whose frequencies depend on the projection of the field along the NV symmetry axis. The setup is integrated with microwave (MW) equipment to monitor NV spin transitions and with a single phonon counter module coupled with a single mode fiber.^30–32^

## 3. Results and discussion

**Fig. 3:**
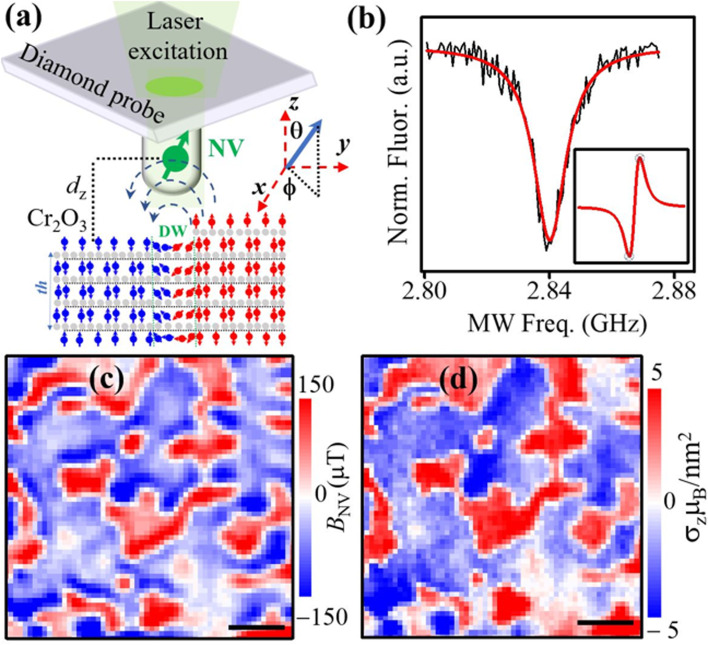
(a) A sketch of NV-SPM with an NV located a distance *d*_*z*_ from Cr_2_O_3_ film surface with opposite magnetic domains separated by a domain wall. Strong magnetic stray fields (dashed field lines) are generated at the DW indicating regions of opposite order parameter defined by the orientation (red up or blue down) of the Cr^3+^ atom. (b) ODMR peak on NV at *B*_A_ = 1.07 mT fitted with Lorentzian function. Insert: zoomed derivative spectrum of an ODMR peak for |*m*_s_ = 0> to |*m*_s_ = −1> transition to select the two MW points needed for *B* dual-iso imaging. (c) *B*_NV_ map of 5 μm Cr_2_O_3_ region (scale bar is 1 μm). (d) The extracted moment density profile of image in (c) which reveals a domain pattern of spin-up and spin-down domains (scale bar is 1 μm).

NV magnetometry probes the stray magnetic field resulting from local surface spins.^19–21^ In noncollinear AFMs, the measured stray field emerges from an overall ferrimagnetic moment due to the canting of the spins.^8,33,34^ However in collinear AFMs such as Cr_2_O_3_, NV magnetometry senses the stray field *B*_NV_ generated from a single layer of uncompensated surface spins, Fig. 3a.^23,25,35^ We performed ODMR measurements on the diamond probe in Fig. 2c by sweeping the MW frequency across the NV |*m*_*s*_ = 0> to |*m*_s_ = −1> transition at an applied field *B*_A_. The Hamiltonian of the system in the lab coordinates (*x*, *y*, *z*) in Fig. 3a is:^18,36^*H* = *DS*_*z*_2 − *γ*_NV_(*S*_*x*_(*B*_A*x*_ + *B*_*x*_) + *S*_*y*_(*B*_A*y*_ + *B*_*y*_) + *S*_*z*_(*B*_A*z*_ + *B*_*z*_))where *D* is the zero-field splitting = 2.87 GHz, *γ*_NV_ = 28 GHz *T*^−1^ is the gyromagnetic ratio of the electron spin. The second term of the Hamiltonian is the Zeeman splitting term. By increasing/decreasing the amplitude of *B*_A_ the resonance of the |*m*_s_ = 0> to |*m*_s_ = −1> transition peak shift to higher/lower frequencies respectively. By monitoring the NV fluorescence increase/decrease, we can measure the magnetic field generated by the magnet *B*_A_ and the additional local stray field *B*_NV_ generated from scanning across the spin textures of Cr_2_O_3_ film. This is the basis of DC magnetic sensing scheme of NV magnetometry.^18–21^ The optimized DC measurable magnetic field in the ideal photon-shot-noise limit is given by:^22,36^*B*_min_ ≅ 4 *Γ* (33 *γ*_NV_ × *C*)^−1^ (*I*_0_ × *t*)^−1^where *Γ* is the full-width-at-half-maximum linewidth of the ODMR peak, *C* is the ODMR peak contrast, *I*_0_ is the NV fluorescence rate, and *t* is the measurements time.^37^ By using the parameters of the NV measurements in Fig. 2c and 3b (*I*_0_ = 500 k counts per s, *Γ* = 7.78 MHz, *C* = 0.06) we found *B*_min_ = 5 μT for *t* = 1 s and a confocal detection voxel of 350 × 350 nm^2^, which is more than sufficient to resolve magnetic stray fields *B*_NV_ from AFM domains in Cr_2_O_3_ (Fig. 3c).

For our measurement we used *B* dual-iso imaging method^38^ to image AFM domains in 200 nm thick Cr_2_O_3_/Al_2_O_3_ substrate. The NV fluorescence is recorded alternatingly at two MW frequencies centered around the ODMR frequency of transition |*m*_s_ = 0> to |*m*_s_ = −1>, chosen at the maxima of the derivative of the measured ODMR peak (crossed dots in the insert of Fig. 3b). By measuring the NV fluorescence (Fl) at each point we can measure directly the signal *S* = Fl(*f*_1_) − Fl(*f*_2_))/(Fl(*f*_1_) + Fl(*f*_2_), which gives positive or negative magnetic stray fields based on the spins orientation of the magnetic domain.^28,39^ This method is suitable for imaging magnetic samples with high (>10 μT) stray fields and allows faster scanning times in comparison to recording the full ODMR spectrum.^40^ The obtained *B*_NV_ image at an applied filed *B*_A_ = 1.07 mT shows two oppositely magnetized domains with areas of positive *B*_NV_ (red) and negative (blue) *B*_NV_, separated by sharp domain walls zeros in *B*_NV_ (white color), Fig. 3c.

In most of surface magnetometry techniques, the inverse problem is used to calculate the magnetization distribution.^21^ In here, we used a similar model to ref. 23 and 41of *B*_NV_ by describing the magnetization *m* of the AFM film as two monolayers of out-of-plane spins (Fig. 2a) with opposite directions, separated by a distance *th* and have a moment density *σ*_*z*_(*x*, *y*):^23^*m* (*x*, *y*, *z*) = *σ*_*z*_(*x*, *y*)[*δ*(*z*) − *δ*(*z* + *th*)]*z*, where *th* is the thickness of the chromia film = 200 nm, *δ* is the Dirac delta function and *z* is the unitary direction. The calculated *B*_NV_ is then obtained by field propagation in the Fourier space:^23,25,41^*B*_NV_(*k⃑*) = *m*(*k⃑*) *T*_NV_ (*d*_*z*_, *θ*_NV_, *ϕ*_NV_, *k⃑*), where *T*_NV_ is a propagator that depends on the NV orientation (*θ*_NV_, *ϕ*_NV_) and the NV-to-sample distance *d*_*z*_ (Fig. 3a). The magnetic moment density profile is determined from the measured *B*_NV_ map using:^23,41^*σ*(*k*) = *T*_NV_^−1^(*d*_*z*_, *θ*_NV_, *φ*_NV_)*W*(*k*)*B*_NV_(*k*), where *W*(*k*) is the filter function given by a Hanning window in the Fourier space.^23^ By using the parameters of our NV geometry (*d*_*z*_ = 50 nm, *θ*_NV_ = 54°, and *ϕ*_NV_ = 92°), we reverse propagated the measured *B*_NV_(*x*, *y*) map in Fig. 3c to calculate *σ*_*z*_(*x*, *y*) map displayed in Fig. 3d. The resulting calculated magnetization image shows the presence of homogeneously magnetized domains, with well-defined domain walls with surface moment density of ∼5 *μ*_B_ nm^−2^. These regions correspond to antiferromagnetically ordered states with opposing orientation of the Néel vector, as sketched in Fig. 3a.

The size of the measured domains varies between 200 nm to 2 μm and it is bigger than the average AFM domain size measured on granulated films (∼230 nm).^23^ We attribute this finding to the fact that our samples have been grown by PLD. Previously,^42^ sister samples grown under identical conditions *via* PLD have been characterized *via* high angle annular dark-field (HAADF) scanning transmission electron microscopy (STEM). The STEM data revealed epitaxial growth of Cr_2_O_3_ (0001) on *c*-plane sapphire. Although PLD grown samples exhibit a new type of planar crystallographic defects on length scales of the order of just a few nanometers, which are comprised of a 60° rotation about the *c*-axis combined with a 1/3 [101̄0] lattice shift,^42^ these defects leave the magnetic exchange interaction between Cr^3+^ ions and the resulting antiferromagnetic order virtually unaffected. Grains and their grain boundaries in sputtered samples behave differently. Here exchange bonds are broken turning grain boundaries into magnetic defects. The abundance of grains in sputtered samples increases the number of domain nucleation sites. With an increased formation of nucleation sites on cooling to below the Néel temperature, the likelihood that two growing antiferromagnetically ordered regions with opposing orientation of the Néel vector meet and form a domain wall increases. With an increasing number of magnetic domains their average size decreases accordingly. This mechanism explains the reduced domain size in sputtered samples with grains on the order of 50 nm,^23^ while also providing a statistical interpretation of the fact that magnetic domains can and typically do grow beyond the size of the grains.

**Fig. 4:**
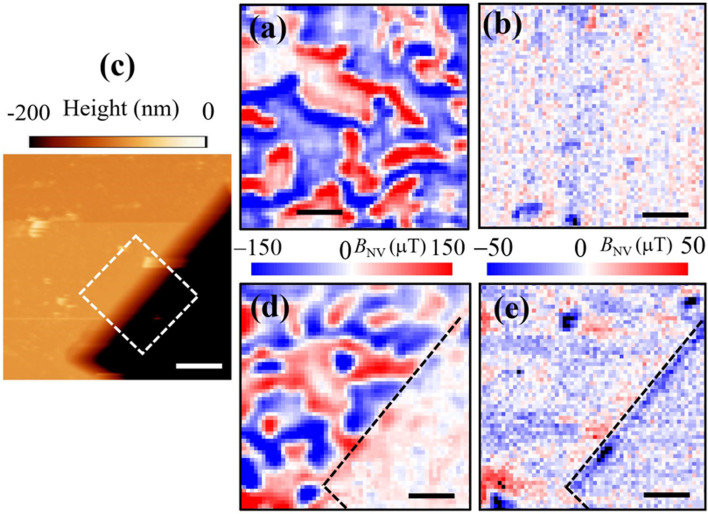
Magnetic field cooling of AFM magnetic domains in chromia. (a) *B*_NV_ map of a given region in chromia film prior to filed cooling. (b) *B*_NV_ map of region (a) after heating above 320 K then cooling to 295 K under a magnetic field of −0.4 T. (c) Atomic force microscope topography map of a zoomed FIB etched nanostructure in Cr_2_O_3_ film. *B*_NV_ map of the region shown in (c) prior (d) and after (e) field cooling under a magnetic field of −0.4 T. Scale bar is 1 μm in (a), (b), (d), and (e).

The antiferromagnetic domain pattern in Cr_2_O_3_ film is strongly affected by the applied magnetic field and temperature conditions, as shown in Fig. 4. To demonstrate this we field-cooled the chromia film (heating to 320 K (>*T*_N_ = 307 K (ref. 12) and cooling to ambient condition (*T* = 295 K) under applied parallel (positive)/anti-parallel (negative) magnetic field *B*_A_ applied along the *c*-axis of the chromia film, Fig. 2d. Prior to field cooling we imaged the film and recorded the *B*_NV_ map in Fig. 4a with a similar domain pattern to Fig. 3c. The result of the field cooling at *B*_A_ = −0.4 T is an ordered spin state with no apparent AFM domains (Fig. 4b). The experimental confirmation of selection of a single domain state confirms that in thin films where parasitic magnetization is present, magnetic field cooling in the absence of an applied electric field can select an antiferromagnetic single domain state with uniform boundary magnetization.^43,44^ Previous experiments were based on integral magnetometry and the presence of uniform boundary magnetization was an interpretation rather than a measured observation. In contrast to polydomain states where higher stray fields are observed in Fig. 4b at the DW edges the *B*_NV_ contrast is low due to the weak stray-field generated from the out-of-plane component of the surface magnetization. This can also be seen at the center of the AFM domains in Fig. 3cand 4a, far away from the DW edges. To obtain a measurable magnetic stray field from uniformly ordered magnetic domains,^35^ we made micron-scale mesas by etching features in Cr_2_O_3_ film grown on Al_2_O_3_ by using focused ion beam (FIB, provided by FEI Helios NanoLab 660). We used a voltage of 30 kV and a current of 7.7 pA to etch 200 nm down the chromia film. We performed scanning electron microscopy (SEM) imaging, not shown here, to monitor the etching and image the etched film regions. Fig. 4cshows the topography atomic force microscope image of a zoomed 5 μm × 10 μm FIB structure with a depth of ∼200 nm. The dark area corresponds to the Al_2_O_3_ substrate. We first imaged the pristine region in Fig. 4cand found the presence of the AFM domains in the unetched region and no apparent contrast is present in the etched region (Fig. 4d) as expected from Al_2_O_3_. We then used a similar field cooling as in Fig. 2d and observed only a magnetic contrast (blue: negative stray field) at the edge of the etched regions, Fig. 4e. Some of the AFM domains in the unetched regions of the film are not switched completely as in Fig. 4b that we explain by the FIB induced defects resulting in pinned magnetic domains.^45^

**Fig. 5:**
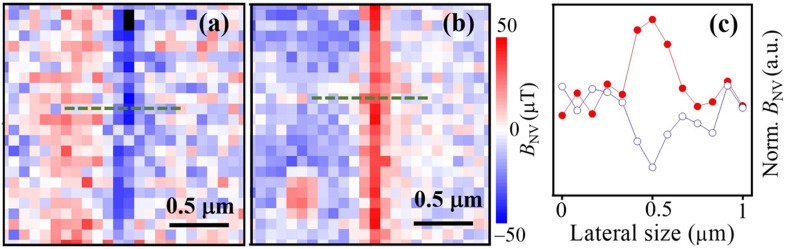
*B*_NV_ map for −0.4 T (a) and +0.4 T (b) field cooled AFM states showing a magnetic field reversible edge DW. (c) Transverse cut of the *B*_NV_ maps in (a) and (b) showing the spatial profile of the edge DW.

The switching of AFM order is clearly seen at the edge of the etched region where the FIB edge defects create a residual edge magnetic domain with a stronger out of plane stray field in comparison to the unetched region (Fig. 4b). The reason of the AFM order switching in the presence of only applied magnetic field is that in thin Cr_2_O_3_ films grown by any method (*e*.*g*., PLD in our case) there is a parasitic magnetic moment that originates from defects in the bulk and the difference in the boundary magnetization between the surface next to vacuum and the surface with the substrate (sketch in Fig. 3a). This magnetic moment is tied to the orientation of the antiferromagnetic order parameter.^15^ Because the magnetic moment orients with the direction of magnetic field on cooling (Fig. 5), the Néel vector orients accordingly. In bulk Cr_2_O_3_ crystals the parasitic moment is very weak, and the Zeeman energy induced by the applied magnetic field is not strong enough to switch the orientation of Néel vector. One need to apply an electric field in addition to the applied magnetic field to activate the magnetoelectric effect and thus creating an energetic favor of one AFM single domain over the other.^12,13,15,35^

To determine the spatial profile of the nanostructured edge AFM state and whether it switches its spin orientation by changing the applied magnetic field direction we performed magnetic field cooling experiments under parallel (positive) and antiparallel (negative) magnetic field applied along the c-axis of the chromia film in the region highlighted (dashed white square) in Fig. 4c. The results are displayed in Fig. 5a and b for antiparallel (−0.4 T) and parallel (+0.4 T) field to the surface of the chromia film respectively. The lateral width of the edge domains is ∼330 nm (Fig. 5c), consistent with the size of the FIB edge shown in Fig. 4c. This makes the width of the edge domain way above the width of DWs (<100 nm) in Fig. 3c and 4a. We explain the magnetic behavior of the edge by the reduction of neighboring spins paired with the creation of defects in the edge region where material has been removed through ion milling. This makes the edge region prone to increased magnetic susceptibility where magnetization is induced with magnetic fields as low as 0.4 T while the rest of the long range ordered sample remains in its more robust AFM state which is known to require significantly higher magnetic field to reverse.^43,44^ The width of this edge region is therefore not a characteristic length scale associated with domain walls formed by competition between anisotropy and intrinsic exchange but rather a fingerprint of lateral extends of damage created by the ion milling process. In contrast to these edge states, domain walls reflect the intrinsic magnetic properties of a sample in conjunction with extrinsic anisotropy contributions such as strain. Our findings regarding DW width away from artificially created edges is very similar to the DW widths (range of 20–100 nm) reported in ref. 25 and 35, depending on the type of the DW (Néel or Block).^25^ This confirms the notion that the DW widths are significantly smaller than expected in the bulk of the low anisotropy magnet Cr_2_O_3_ and determined by epitaxial strain.^46^ Recent NV measurements on DWs created *via* magnetoelectric field cooling in Cr_2_O_3_ single crystals revealed DW-pinning phenomena at mesa edges engineered by electron beam lithography.^35^ The observed interaction is explained by the competition between surface energy of the domain wall.

Of notice is the spatial resolution of our NV-SPM microscope (∼50 nm), as determined from the magnetization reconstruction of *B*_NV_ map in Fig. 3d, and thus high enough to measure sub-50 nm DWs. We explain the degradation of the spatial resolution from <15 nm to ∼50 nm by the large amplitude of the diamond tip oscillation and low Q-factor of the tuning fork cantilever at ambient conditions.^20,21,25^ Integrating NV-SPM with ultra-high vacuum system will enhance the Q-factor of the diamond probe by orders of magnitude^20,47^ and allow imaging AFM spin textures with a spatial resolution below 15 nm, defined mainly by the distance from the NV tip.^20,21,28,39^

## 4. Conclusions

In summary, NV stray-field imaging measurements on magnetic field cooled epitaxial Cr_2_O_3_ films revealed switching of the AFM order to just one magnetic state we explained by the presence of a parasitic magnetic moment, tied to Néel vector. The domain formation in PLD grown samples is different from the domain formation in sputtered samples where structural grains act as nucleation centers giving rise to an increased number of domains with reduced size. At the same time, the domain wall width seems to be unaffected by the growth method and is primarily controlled by a narrowing mechanism caused by lattice strain. The lattice strain depends on the mismatch between substrate and thin film and, hence is identical for all Cr_2_O_3_ thin films grown on *c*-plane sapphire. Furthermore by nanostructuring the chromia film we created edge states that switch its orientation based on the direction of applied field during field cooling experiments. These results could motivate further investigations of DW engineering in antiferromagnets.^4,17,25,35^ For example boron doping in epitaxial Cr_2_O_3_ films increases *T*_N_ to 400 K^48^ and enables voltage control of AFM order and Néel vector orientation at zero applied magnetic field.^49^ In such structures the AFM spin textures are not yet explored at the nanoscale.

## Author contributions

The manuscript was written through contributions of all authors. All authors have given approval to the final version of the manuscript. AE performed NV-SPM measurements and analyzed the data; ASQ, AM grew Cr_2_O_3_ films and performed topography and XRD measurements; RT assisted AE in FIB and SEM measurements; IF wrote the Mathematica code to get the magnetization profile from NV stray field measurements. AL and CB designed the experiments and supervised the project.

## Conflicts of interest

The authors declare no competing financial interest.

## Supplementary Material
