# Correction: Nanoscale imaging of antiferromagnetic domains in epitaxial films of Cr_2_O_3_*via* scanning diamond magnetic probe microscopy

**DOI:** 10.1039/d3ra90001k

**Published:** 2023-01-16

**Authors:** Adam Erickson, Syed Qamar Abbas Shah, Ather Mahmood, Ilja Fescenko, Rupak Timalsina, Christian Binek, Abdelghani Laraoui

**Affiliations:** a Department of Mechanical & Materials Engineering, University of Nebraska-Lincoln 900 N 16th St., W342 NH Lincoln Nebraska 68588 USA alaraoui2@unl.edu; b Department of Physics and Astronomy and the Nebraska Center for Materials and Nanoscience, University of Nebraska-Lincoln 855 N 16th St Lincoln Nebraska 68588 USA binek@unl.edu; c Laser Center, University of Latvia Jelgavas St 3 Riga LV-1004 Latvia

## Abstract

Correction for ‘Nanoscale imaging of antiferromagnetic domains in epitaxial films of Cr_2_O_3_*via* scanning diamond magnetic probe microscopy’ by Adam Erickson *et al.*, *RSC Adv.*, 2023, **13**, 178–185, https://doi.org/10.1039/D2RA06440E

The authors regret that there were errors that appear in the Results and discussion section. On line 47 in the right column on page 179, the text originally read “*H* = *DS*_z_2 − *γ*_NV_(*S*_x_(*B*_Ax_ + *B*_x_) + *S*_y_(*B*_Ay_ + *B*_y_) + *S*_z_(*B*_Az_ + *B*_z_))”. It should read “*H* = *DS*_z_^2^ − *γ*_NV_(*S*_x_(*B*_Ax_ + *B*_x_) + *S*_y_(*B*_Ay_ + *B*_y_) + *S*_z_(*B*_Az_ + *B*_z_))”. On line 7 in the left column on page 180 of the original article, the text originally read “*B*_min_ ≅ 4 *Γ* (33 *γ*_NV_ × *C*)^−1^ (*I*_0_ × *t*)^−1^”. It should read “*B*_min_ ≅ 4 *Γ* (3√3 *γ*_NV_ × *C*)^−1^ (*I*_0_ × *t*)^−1/2^”. On line 23 in the left column on page 180 of the original article, the text originally read “*S* = Fl(*f*1) − Fl(*f*2))/(Fl(*f*1) + Fl(*f*2)”. It should read “*S* = (Fl(*f*1) − Fl(*f*2))/(Fl(*f*1) + Fl(*f*2))”.

The authors regret that there was an error in the text in the Author contributions section on line 24 in the right column on page 183 of the original article. The text originally read “ASQ”. It should read “SQAS”.

The Royal Society of Chemistry apologises for these errors and any consequent inconvenience to authors and readers.

## Supplementary Material

